# Fatigue Life Evaluation and Structural Optimization of Rubber Damping Components in Metro Resilient Wheels

**DOI:** 10.3390/polym18080915

**Published:** 2026-04-09

**Authors:** Qiang Zhang, Zhiming Liu, Yiliang Shu, Guangxue Yang, Wenhan Deng

**Affiliations:** 1 School of Mechanical and Electronic Control Engineering, Beijing Jiaotong University, Beijing 100044, China; 22115118@bjtu.edu.cn (Q.Z.); zhmliu1@bjtu.edu.cn (Z.L.); gxyang@bjtu.edu.cn (G.Y.); 2 Shanxi Taibao Sealing Technology Co., Ltd., Taiyuan 030032, China; 3 School of Mechatronics and Vehicle Engineering, East China Jiaotong University, Nanchang 330199, China; 4 BYD Company Limited, Shenzhen 518118, China; 22126085@bjtu.edu.cn

**Keywords:** resilient wheel, rubber vibration-damping components, structural optimization, fatigue life prediction

## Abstract

Resilient wheels are widely employed in metro vehicles to mitigate vibration and noise, in which rubber damping components play a critical role in load transmission and fatigue resistance. However, stress concentration and cyclic loading can significantly compromise their durability and service life. In this study, the structural optimization and fatigue life of rubber damping components in resilient wheels are systematically investigated based on finite element analysis and in-service metro operational data. A three-dimensional finite element model incorporating hyperelastic material behavior is developed to evaluate stress distributions under three representative conditions: press-fit assembly, straight-line operation, and curved-track operation. Based on the resulting stress fields, critical high-stress regions within the rubber component are identified and selected as targets for structural optimization. The Design of Experiments (DOE) methodology, integrated with the Isight 2022 optimization platform, is employed to determine the optimal geometric parameters that minimize the von Mises equivalent stress. Furthermore, a fatigue life prediction framework is established using actual metro service mileage data. Fatigue performance is assessed using Fe-safe 2022 software in conjunction with rubber fatigue crack propagation theory, and the results before and after optimization are systematically compared. This study demonstrates that stress concentrations in resilient wheel rubber damping components predominantly occur at fillet transition regions, governed by load transfer characteristics under press-fitting and service conditions. Through DOE-based structural optimization, the critical geometric parameters are effectively refined, leading to a significant reduction in stress levels in key regions. As a result, the proposed approach markedly improves fatigue performance, extending the minimum fatigue life from 1300 days to 24,322 days, thereby substantially enhancing the durability and reliability of the resilient wheel system.

## 1. Introduction

Rubber is widely utilized in engineering applications owing to its outstanding properties, including high elasticity, large deformation capability, and near-incompressibility. Consequently, it is extensively employed in the manufacture of components such as tires, vibration isolators, and sealing elements, with broad applications spanning aerospace and industrial systems. Rubber fatigue refers to the progressive degradation of rubber structural components subjected to cyclic uniaxial or multiaxial loading during service. Under such loading conditions, damage accumulates in the form of microcrack initiation and propagation, leading to stiffness degradation and, ultimately, localized fracture in regions of stress concentration. Therefore, the fatigue resistance of rubber components plays a decisive role in determining their service performance and structural reliability. A thorough understanding of fatigue mechanisms, together with accurate prediction of the service life of rubber structures under realistic operating conditions, is of significant practical importance for ensuring the safety and durability of engineering systems.

**Figure 1:**
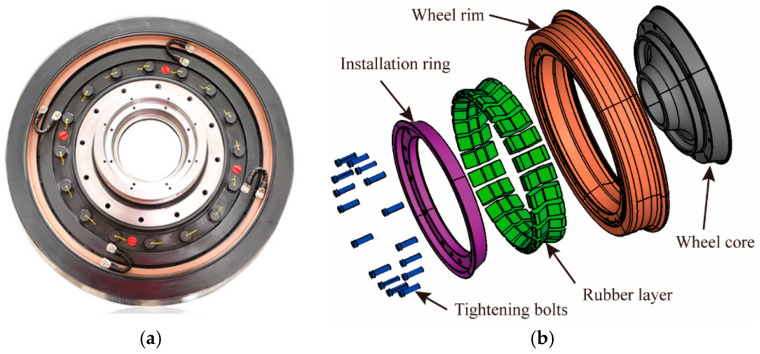
Physical specimen of resilient wheel and its key components: (**a**) Physical specimen diagram; (**b**) exploded view diagram.

As a critical load-bearing structure in urban rail transit vehicles, the installation of elastic rubber components between the wheel rim and the wheel center of resilient wheels enhances the vibration-damping and noise-reduction performance of the vehicle. Research indicates [1] that the use of resilient wheels mitigates wheel–rail impact noise, attenuates wheel–rail vibration, and reduces dynamic stresses in the bogie transmission system. During operation, vibrations and noise generated by wheel–rail contact in urban rail transit are inevitable. When trains pass through curves, turnouts, or experience strong wheel–rail impacts, these vibrations and noise significantly affect operational stability and passenger comfort [2,3]. The resilient wheel, as illustrated in Figure 1, consists of a rubber layer integrated with the wheel core/web, wheel rim, installation ring, and bolts. When assembled with the axle to form a wheelset, this configuration serves as a key solution for vibration and noise mitigation in urban rail transit systems. The inherent damping properties of the rubber layer in resilient wheels substantially reduce wheel–rail contact loads [4]. Press–shear composite resilient wheels demonstrate superior mechanical performance compared with purely pressed or purely sheared resilient wheels. Therefore, press–shear composite resilient wheels are widely used in urban rail transit systems [1,5]. However, since resilient wheels are continuously subjected to complex dynamic loads, environmental temperature variations, and frictional interactions between components during service, the internal rubber vibration-damping elements are prone to fatigue damage. This degradation reduces the vibration-damping and noise-reduction effectiveness of the wheels and may lead to severe safety issues in rail vehicles. Hence, studying and predicting the fatigue life of resilient wheel rubber components is essential for ensuring the operational safety and reliability of rail transit systems.

Rubber materials exhibit a wide range of types, and factors such as filler composition, processing techniques, and operating environments influence their physical properties. Moreover, rubber components are often subjected to time-varying multiaxial fatigue loads during service, leading to complex time-dependent stress–strain behavior and variations in mechanical parameters within rubber structures. This complexity makes it challenging to evaluate fatigue performance, investigate failure mechanisms, and accurately predict the fatigue life of rubber structures. Research on the fatigue behavior of rubber structures first emerged in the 1940s. Since then, numerous life prediction models for rubber materials and structures have been proposed; however, no unified predictive framework capable of accurately estimating the fatigue life of rubber components has yet to be established. Furthermore, studies on the mechanisms of rubber fatigue have not reached consistent conclusions [6,7,8]. Methods for assessing rubber fatigue performance and predicting service life are generally classified into crack initiation approaches, crack propagation methodologies, and artificial neural network (ANN) models [9,10,11,12,13,14]. Crack initiation methods aim to establish relationships between fatigue damage parameters and rubber fatigue life, while crack propagation approaches primarily investigate the correlation between tear energy and crack growth. The use of ANN-based models to predict rubber fatigue life represents a novel approach developed in recent years, offering high predictive accuracy. The crack propagation process in rubber typically consists of two stages: crack initiation and crack propagation. During the initiation stage, the rubber material shows no visible cracks; under external cyclic loads, microscopic defects gradually initiate and evolve into cracks. During the propagation stage, internal cracks continue to grow under alternating loads until localized damage or complete structural fracture occurs [15]. Cadwell [16] pioneers the use of crack initiation methods to evaluate rubber fatigue life. Shangguan et al. [17] report from uniaxial tensile fatigue tests that crack initiation accounts for more than 90% of the total fatigue life, suggesting that the fatigue crack initiation life can be approximately equated with the overall fatigue life of rubber. Li et al. [18] conduct uniaxial fatigue tests using maximum principal strain as the fatigue damage parameter and find good agreement between predicted and experimental lifetimes. Belkhiria et al. [19] develop fatigue life prediction models for rubber materials based on logarithmic strain, engineering strain, Green–Lagrange strain, Euler strain, and octahedral shear strain, and conclude that strain-based parameters effectively describe rubber fatigue life, with the octahedral shear strain model providing the highest prediction accuracy. All these experimental results are obtained under uniaxial fatigue conditions and yield favorable life predictions. However, uniaxial fatigue conditions cannot be directly applied to predict the fatigue life of rubber structural components under complex multiaxial loading. Ayoub et al. [20] compare different fatigue damage parameters for predicting rubber fatigue life under multiaxial loading and find that the maximum principal strain fails to accurately predict both uniaxial and multiaxial fatigue life simultaneously. Saintier et al. [21] employ the first and second invariants of Cauchy stress as fatigue criteria to predict rubber multiaxial fatigue life, but their results indicate that stress-based criteria cannot accurately predict multiaxial fatigue behavior. Poisson et al. [22] compare uniaxial and multiaxial fatigue test results and conclude that the first principal stress does not reliably predict fatigue life. Wang et al. [23] establish a computational model for predicting uniaxial tensile fatigue life based on continuum damage mechanics, achieving high prediction accuracy. Ayoub et al. [24,25] propose an effective stress parameter derived from damage mechanics and crack energy density, which enables a relatively accurate prediction of multiaxial fatigue life in rubber materials.

Crack propagation methods represent an approach to studying the fatigue life of rubber based on fracture mechanics. This methodology assumes that microcracks inevitably form within rubber structures due to manufacturing processes and inherent material defects. Lake and Lindley et al. [26,27] conducted crack propagation experiments on rubber materials, dividing the crack growth process into four distinct stages and establishing fatigue life prediction models corresponding to each stage. Asare et al. [28] investigated the fatigue crack propagation behavior of rubber materials at both ambient and elevated temperatures based on fracture mechanics theory, confirming the validity of this approach for evaluating rubber fatigue life. Ait-Bachir et al. [29] demonstrated that the energy release rate of small cracks is proportional to crack size and independent of the loading environment and crack orientation. Fukahori et al. [30] revealed the transition relationship between the critical strain energy release rate and the critical crack propagation rate, based on the elastic–viscoelastic transition behavior observed in rubber. Although crack initiation and propagation methods effectively simulate rubber fatigue failure and enable fatigue life prediction, these approaches require extensive experimental data and complex numerical derivations, leading to high computational costs and limited practical applicability. Data-driven methods utilize machine learning techniques to analyze experimental data and establish nonlinear relationships between inputs and outputs, thereby enabling the prediction of rubber fatigue life. Wang et al. [31] proposed a support vector machine (SVM) model that uses engineering strain amplitude, engineering strain mean, and strain ratio as input variables, and measured fatigue life as the output variable, to predict the fatigue life of rubber under different strain ratios. Liu et al. [32] developed an artificial neural network (ANN) model employing peak engineering strain, ambient temperature, and material hardness as inputs, with measured fatigue life as the output, to predict rubber fatigue life under varying temperature and hardness conditions. To improve the predictive accuracy of data-driven models, integrating the theoretical rigor of physics-based models with the adaptability of data-driven approaches yields a more accurate and practical method: the physics-informed neural network (PINN) model. Halamka et al. [33] and Wang et al. [34] proposed PINN frameworks that combine physical knowledge of material behavior—such as stress–strain relationships and crack propagation rates—with ANN architectures, thereby enhancing the predictive accuracy of fatigue life estimation for metallic materials.

Although extensive fatigue testing and life prediction analyses of rubber materials and structures are conducted by researchers worldwide, relatively few studies focus on predicting the fatigue life of rubber blocks within resilient wheels used in rail transit. Current research in the rail transit field primarily concentrates on the vibration damping, noise reduction, and dynamic performance of resilient wheels [35,36,37,38,39,40,41,42,43], while studies on the fatigue life prediction and structural optimization of their internal rubber damping components remain limited. The fatigue life of rubber damping components directly affects the overall fatigue performance of resilient wheels and consequently influences the service performance and operational safety of the entire vehicle system. Therefore, it is essential to establish a fatigue life prediction method for the internal rubber damping components of resilient wheels. Such a method enables the quantitative assessment of fatigue damage, supporting the optimization of the service life cycle and maintenance intervals of resilient wheels. To evaluate the service life of rubber vibration-damping components in resilient wheels, it is necessary to develop a hyperelastic constitutive model for these components and to determine their key material parameters. This allows the application of finite element (FE) numerical methods to analyze the fatigue life of rubber components.

In research on performance prediction and damage diagnosis of rubber materials, the innovative application and optimization of machine learning methods have become a key breakthrough. Zhang et al. [44] adopted a Stacking ensemble learning framework that integrates base models such as Random Forest (RF) and K-Nearest Neighbors (KNN), effectively improving the robustness of corrosion degree prediction in rubber concrete and addressing the insufficient anti-interference capability of a single model. Choudhury et al. [45] focus on the prediction of rubber tensile strength and systematically compare the performance of Decision Tree (DT), RF, and Extreme Gradient Boosting (XGBoost) models, demonstrating that the RF model achieves the best predictive performance due to its ability to accurately capture the nonlinear relationships between composition and properties. Shen et al. [46] addressed the bottleneck of small-sample modeling by optimizing the number of components in a Gaussian Mixture Model using the AIC/BIC criteria, generating reasonable virtual samples and providing effective support for model training under data-scarce conditions. He et al. [47] standardized the composition and strength data of rubber concrete, eliminating the influence of dimensional differences on model training and offering a standardized data preprocessing scheme for modeling. Deng et al. [48] proposed an innovative hybrid model (1DCNN-LSTM-BO-XGB), in which 1DCNN extracts local features from vibration signals of rubber bearings, LSTM captures temporal dependencies, and an XGBoost model optimized by Bayesian Optimization (BO) is employed for damage detection. This approach achieves an outstanding accuracy of 98.6% in detecting six levels of damage in rubber bearings, significantly outperforming individual models such as 1DCNN (88.9%), LSTM (25.0%), and XGBoost (90.3%), thereby highlighting the superiority of hybrid models in complex tasks. Therefore, the development trend of combining rubber materials with machine learning evolves from the application of single base models toward ensemble learning and hybrid modeling that integrates deep learning with traditional machine learning approaches.

The authors’ research group prepares compression specimens based on the material composition of the resilient wheel rubber components and conducts compression tests to obtain force–displacement data. The key parameters of the hyperelastic constitutive model are then determined by integrating FE simulation results with deep learning methodologies [49]. Although the fatigue life of rubber structural components is directly influenced by filler type, service conditions, and processing techniques, this study specifically considers carbon black as the reinforcing filler. The content and dispersion state of carbon black directly determine the density and crosslinking degree of the rubber network structure. An increase in carbon content enhances the network density, but excessive filler may form agglomerates that act as crack initiation sites, significantly accelerating crack propagation. High-frequency cyclic loading exacerbates viscoelastic hysteresis and self-heating in the rubber, while elevated operating temperatures further reduce the stability of the crosslinked network. These combined effects accelerate network degradation and crack growth, demonstrating a clear link between service conditions and fatigue life. Processing techniques influence filler dispersion uniformity and the integrity of the crosslinked network, which in turn affect the mechanical performance and fatigue resistance of the rubber component. Improper processing parameters may result in locally uneven crosslinking, creating stress-weak regions that serve as preferential crack propagation paths. Therefore, the fatigue life of resilient wheel rubber damping components results from the combined influence of multiple factors. In this study, under a given filler type, service load, and processing technique, the focus is on elucidating the effect of the rubber component’s structural dimensions and its interaction with the metallic components of the resilient wheel on fatigue life prediction and structural optimization.

The crack initiation method for rubber fatigue shows certain limitations in accurately predicting multiaxial fatigue life, while artificial intelligence approaches such as neural networks require large amounts of experimental data. Fatigue testing is expensive, and the existing literature provides a limited experimental database. Therefore, this study adopts a rubber fatigue crack propagation approach for fatigue life prediction. Based on the Thomas crack propagation model, the relationship between tear energy and crack propagation rate is established [50], which enables press-fit stress analysis and fatigue life prediction of the rubber vibration-damping element in resilient wheels. ABAQUS 2022–Isight 2022 software is employed to optimize the structure of the resilient wheel’s rubber vibration-damping component, resulting in an optimized configuration for the critical stress regions. By integrating actual track measurement data and real operational conditions, a comparative fatigue life analysis is performed for the resilient wheel before and after structural optimization. The results indicate that the structural optimization significantly enhances the fatigue resistance of the resilient wheel rubber vibration-damping component.

## 2. Numerical Simulation Methods for Rubber Components

The material composition of the resilient wheel rubber damping component consists of ethylene–propylene–diene monomer (EPDM) rubber, zinc oxide, carbon black, stearic acid (SA), dicumyl peroxide (DCP), paraffin oil, antioxidants RD and MB, and accelerator CZ. The rubber compounding process is as follows: The EPDM base rubber is first milled on an open two-roll mill until a homogeneous master batch is obtained. Once the rubber surface is smooth, zinc oxide, carbon black, stearic acid (SA), paraffin oil, antioxidants RD and MB, dicumyl peroxide (DCP), and accelerator CZ are sequentially added. The compound is sheared and folded in triangular packages 5–6 times, the roll gap is then increased to 3 mm, and the compound is rolled five times before being sheeted. The compounded rubber is allowed to rest for one day, and the curing curve is determined using a rheometer. The product processing procedure is as follows: The compounded rubber is first milled on the two-roll mill approximately five times. The rubber is then cut according to the calculated product weight and shape. The cut pieces are placed into molds and molded using a flat platen press. The primary curing conditions are 170 °C for 30 min under a pressure of 15 MPa, followed by a secondary curing step at 150 °C for 4 h.

**Figure 2:**
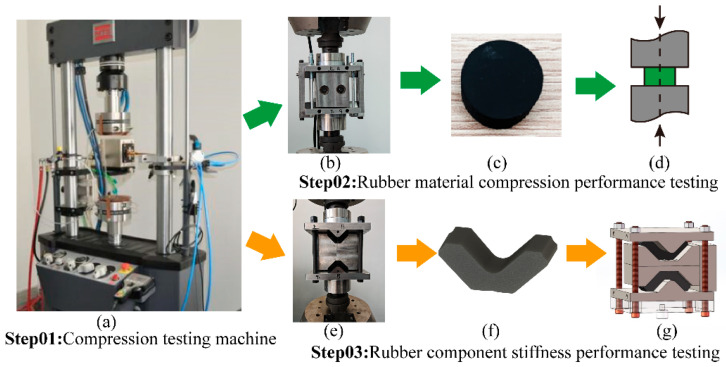
Rubber material and rubber component experiment test (**a**) Overall view of the testing machine; (**b**) Test fixture for cylindrical specimens; (**c**) Rubber cylindrical specimen; (**d**) Schematic diagram of cylindrical specimen loading; (**e**) Test fixture for rubber components; (**f**) Model of rubber component; (**g**) Schematic diagram of rubber component loading.

Rubber components constitute the core structural elements within resilient wheels, functioning to attenuate vibration and reduce noise. On the one hand, these rubber elements suppress vibrations generated during rail vehicle operation, thereby reducing the vibrational energy transmitted to the resilient wheel. On the other hand, the rubber components absorb vibration-induced noise from the bogie or axle system, consequently mitigating noise transmission from the rail vehicle. The physical configuration of a resilient wheel is illustrated in Figure 1a. It primarily consists of a wheel core, rim, rubber layer, mounting ring, and preload bolts, as shown in Figure 1b. As a critical structural component of the resilient wheel, the hyperelastic material constitutive parameters of the rubber vibration-damping element are determined through experimental testing and finite element (FE) numerical simulations. Experimental investigations are conducted using a testing machine to characterize the compressive behavior of the rubber material and to evaluate the stiffness of the rubber damping component, respectively, as shown in Figure 2.

**Figure 3:**
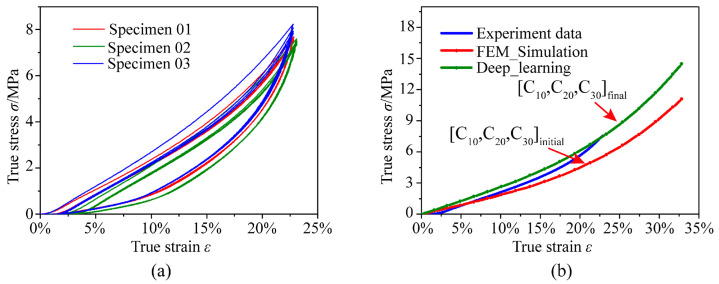
Determination of Yeoh model constitutive parameters: (**a**) Experimentally obtained stress–strain data; (**b**) parameter optimization using a deep learning approach.

The key parameters of the Yeoh model in this study are determined based on force–displacement data obtained from compression tests. Specifically, three specimens are tested, each subjected to three loading cycles, and the averaged results are used to derive the corresponding true stress–strain data, as shown in Figure 3a. Based on the experimental stress–strain data and the theoretical stress–strain relationships for the compression process, the initial Yeoh model parameters *C*_10_, *C*_20_, and *C*_30_ are identified. Subsequently, a finite element model is established to replicate the compression process, and the initial parameter set [*C*_10_, *C*_20_, *C*_30_]_initial_ is implemented. However, discrepancies are observed between the simulated stress–strain response and the experimental results. To improve the accuracy of the model, a parametric study is conducted by systematically varying *C*_10_, *C*_20_, and *C*_30_ over a sufficiently wide range. The corresponding stress–strain responses are obtained through finite element simulations, thereby constructing a comprehensive database with stress–strain data as inputs and material parameters as outputs. A deep learning approach is then employed to train this dataset. Using the experimentally obtained stress–strain data as input, the trained model predicted the optimized Yeoh parameters [*C*_10_, *C*_20_, *C*_30_]_final_. A comparison of the experimental stress–strain data, the response predicted using the initial parameters [*C*_10_, *C*_20_, *C*_30_]_initial_, and that obtained using the optimized parameters [*C*_10_, *C*_20_, *C*_30_]_final_ is presented in Figure 3b. Specific parameter calibration methods can be referenced from previous research works [44]. Using the established constitutive parameters of the hyperelastic model, FE numerical simulation methods are applied in conjunction with existing rail vehicle standards to analyze stress variations in the rubber damping element during the press-fitting process. This approach also investigates the stress–strain behavior of the rubber damping element within the resilient wheel under operational loads, providing a theoretical and computational foundation for optimizing its structural dimensions and predicting its service life.

### 2.1. Numerical Model for Resilient Wheels

**Figure 4:**
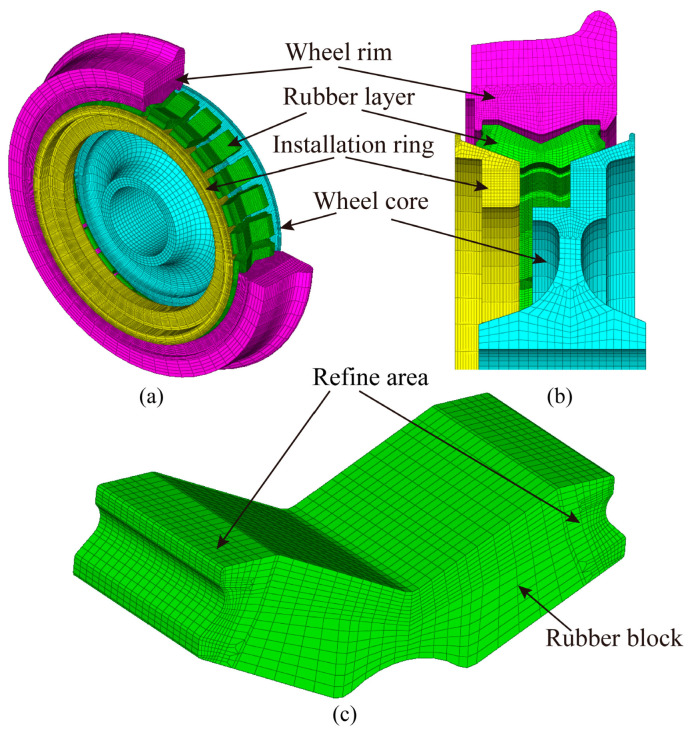
Resilient wheel mesh model: (**a**) Overall model view; (**b**) fitting section view; (**c**) rubber block structured mesh.

The prerequisite for performing finite element (FE) numerical analysis of resilient wheels is the discretization of their three-dimensional geometry to generate a mesh model suitable for FE computation. The three-dimensional geometric model of the resilient wheel is imported into general-purpose FE pre-processing software for mesh generation. The hub, rim, compression ring, and rubber layer—comprising 26 circumferentially arranged and evenly spaced rubber blocks—are each discretized. A structured meshing approach is employed to generate a hexahedral mesh for the resilient wheel. The overall FE model of the resilient wheel is illustrated in Figure 4a, while the pre-press-fit mating relationships of key components are shown in Figure 4b. Before conducting FE simulations of the resilient wheel under service loads, the press-fitting process of each component is simulated. The three-dimensional FE mesh model of a single rubber vibration-damping element is presented in Figure 4. A structured meshing technique is used to discretize the rubber block into a regularly arranged hexahedral mesh. In addition, a transition meshing technique is applied on both sides of the rubber block, locally refining the mesh at the filleted transition regions. This meshing strategy ensures an optimal balance between high computational accuracy and low computational cost.

The finite element (FE) simulation of the resilient wheel is performed using the commercial software ABAQUS 2022. The wheel hub, rim, and pressure ring are modeled with eight-node three-dimensional solid elements with reduced integration (C3D8R). As the rubber component exhibits hyperelastic behavior, each rubber block is modeled using eight-node three-dimensional hybrid solid elements with reduced integration (C3D8RH) to accurately capture its mechanical response. To investigate the influence of mesh density on the computational results, five mesh schemes with different element sizes are designed. In all cases, the geometric parameters, boundary conditions, material constitutive models, and loading conditions remain identical, with only the mesh size being varied. The critical region of interest—namely the arc transition zone between the rubber damping component of the resilient wheel and the contacting metal parts—is selected for local mesh refinement (Refine area in Figure 4), while relatively coarser meshes are applied in non-critical regions to improve computational efficiency. The total number of elements in the five schemes is approximately 355,620, 415,620, 472,180, 542,040, and 592,140, respectively, corresponding to element sizes in the refined regions of 2.0, 1.5, 1.0, 0.5, and 0.25. As the number of elements increases from 200,000 to 500,000, the variation in key evaluation metrics gradually decreases. When the mesh density is further increased from 542,040 to 592,140 elements, the variation rates of the maximum von Mises equivalent stress, maximum strain, and contact pressure are all less than 2%, indicating that mesh convergence is achieved and further refinement has a negligible effect on the results. Based on the above analysis, a mesh scheme with approximately 542,040 elements is adopted in this study, as it ensures sufficient computational accuracy while maintaining reasonable computational efficiency. The complete FE mesh of the resilient wheel consists of 542,040 elements and 612,879 nodes.

The hyperelastic behavior of the rubber block is described using the Yeoh constitutive model, with key material parameters referenced from published research [44]. The resilient wheel consists of the rim, hub, pressure ring, and rubber layer, and load transfer among these components is achieved by defining contact pairs in the FE model. Three contact pairs are established: the rubber layer with the rim, the rubber layer with the core, and the rubber layer with the pressure ring. The contact surface of the rubber layer is defined as the secondary surface, while the contact surfaces of the rim, core, and pressure ring are defined as the primary surfaces. All contact pairs employ the finite sliding contact algorithm. The surface discretization method adopts a surface-to-surface contact formulation, with the contact follower surface set to “No adjustment.” Normal contact behavior between components is modeled using the default “hard” contact formulation, whereas tangential contact behavior is governed by the penalty friction method.

### 2.2. Press-Fitting Processes Numerical Simulation

**Figure 5:**
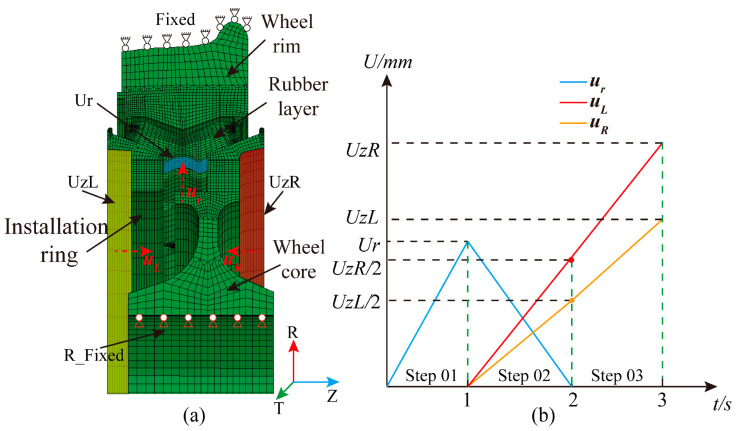
Simulated model of resilient wheel press-fitting: (**a**) Schematic diagram of model loading; (**b**) sequence diagram of displacement load application.

Before applying the vertical and lateral operational loads specified for resilient wheels, press-fit simulations are performed on each component of the wheel. This establishes the pre-tensioned state of the initially compressed rubber layer, enabling a more accurate representation of the stress–strain response of the resilient wheel under service conditions. The resilient wheel mesh model is imported into the finite element software ABAQUS 2022, load constraints are applied to each component, and numerical solutions are computed. The boundary conditions for the press-fit simulation and the displacement loading sequence are illustrated in Figure 5.

In the first step of the press-fit simulation, full constraints are applied to the outer surface of the rim, including the wheel tread, while radial constraints are imposed on the inner bore surface of the hub. An outward radial displacement *U_r_* = 10 mm is applied to the lower surface of the rubber layer. In the second analysis step, an axial displacement *U*_zL_/2 is applied to the outer surface of the press ring, and an axial displacement *U*_zR_/2 is applied to the hub. Finally, in the third analysis step, an axial displacement *U*_zL_ = 20 mm is applied to the press ring, and an axial displacement *U*_zR_ = 27 mm is applied to the wheel hub. The boundary conditions for the resilient wheel press-fit simulation are shown in Figure 5a, while the displacement loading sequence diagram for the resilient wheel is depicted in Figure 5b.

### 2.3. Operational Conditions Numerical Simulation

Considering that the simulation of the press-fitting process and in-service operating conditions of the resilient wheel involves multiple contact interactions and the hyperelastic nonlinear behavior of rubber components, the numerical solution is characterized by strong coupling in convergence and relatively high computational cost. For the press-fitting process, the primary objective of the numerical simulation is to reproduce the assembled state of the resilient wheel. Therefore, by accurately modeling the interaction among the rubber layer, wheel rim, wheel center, and retaining ring in accordance with the actual press-fitting procedure, the resulting post-assembly stress state of the wheel can be reasonably captured. Under this premise, the assumptions adopted in the press-fitting simulation are considered to be appropriate for engineering analysis. For the operational loading conditions, the assembled resilient wheel is subjected to vertical and lateral loads defined according to relevant standards (EN 13979-1 [51] and UIC 510-5 [52]). By applying these standardized load cases, the simulated service conditions can be regarded as representative and sufficiently accurate for evaluating the mechanical response of the wheel during operation.

The wheel–rail contact loads experienced by resilient wheels during actual track operation are primarily classified into straight-line and curved-track conditions. Based on the load definitions for straight-line and curved-track conditions specified in standards EN 13979-1 and UIC 510-5, the corresponding load values applied to resilient wheels are calculated for each condition. The loads on the wheels are directly related to the axle load of the tram. For the tram considered in this study, the axle load is set at 7.8 t, resulting in a load *P* of 3.9 t applied to each wheel. The load calculations for straight-line and curved-line conditions are shown in Equations (1) and (2), enabling the corresponding load values to be determined. The load values calculated according to the Equations (1) and (2) are listed in Table 1.

(1) Linear operating load:

(1) $$\left\{\begin{array}{l} F_{z1}=1.25P\\ F_{y1}=0 \end{array}\right.$$

(2) Curved-line operating load:

(2) $$\left\{\begin{array}{l} F_{z2}=1.25P\\ F_{y2}=0.7P \end{array}\right.$$

**Table 1:** Loads under different operating conditions for resilient wheels.

Operating Condition	Vertical Load/N	Lateral Load/N
Linear operating condition	*F_z_*_1_ = 48,750	0
Curved operating condition	*F_z_*_2_ = 48,750	*F_y_*_2_ = 27,300

**Figure 6:**
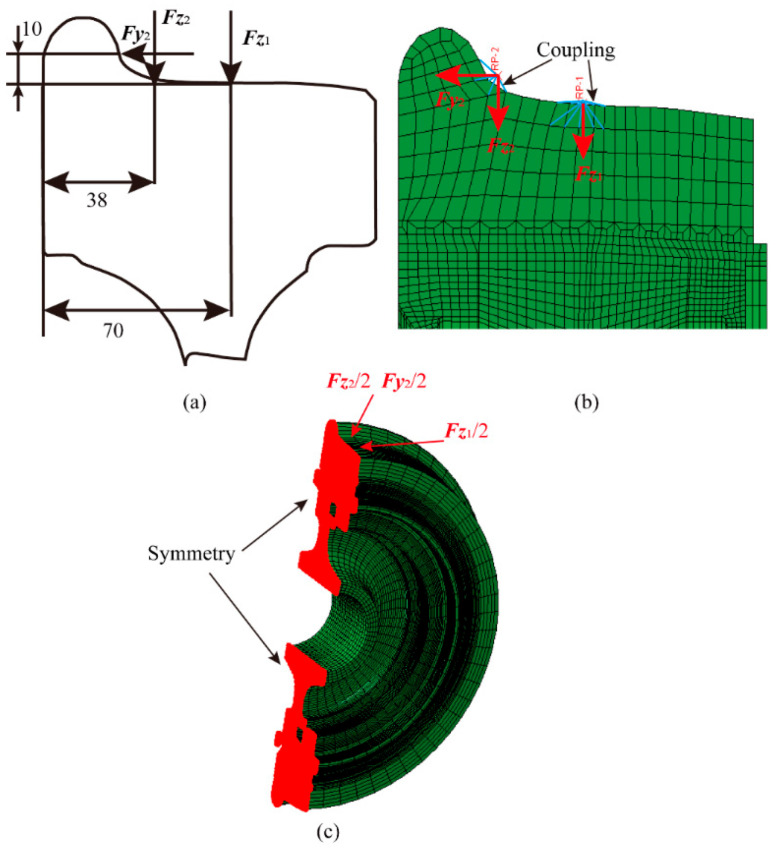
Operational simulation model for resilient wheels: (**a**) Standard-specified loads; (**b**) simulation load application method; (**c**) semi-symmetrical model of resilient wheels.

When applying vertical and lateral loads to the finite element numerical model of a resilient wheel, the precise loading positions must be determined in accordance with standards EN 13979-1 and UIC 510-5. When applying linear loading conditions to resilient wheels that have undergone the press-fitting process, the vertical load *F_z_*_1_ is applied at the tread position 70 mm from the left-hand section of the wheel rim (i.e., at the nominal rolling circle position), as shown in Figure 6a. When applying loads under curved operating conditions, the vertical load *F_z_*_2_ and lateral load *F_y_*_2_ are applied respectively at the tread position 38 mm from the left-hand end face of the wheel rim and at the tread position 10 mm from the vertical distance of the nominal rolling circle, as shown in Figure 6a.

When applying loads corresponding to straight-line and curved-track operating conditions on the tread surface of a resilient wheel, reference points 1 (RP1) and 2 (RP2) are established to avoid stress concentrations or singularities at the loading locations. Coupled constraints are employed to create rigidly connected relationships between the reference points and adjacent nodes, thereby simulating the application of loads under both straight-line and curved-track conditions, as illustrated in Figure 6b. Full constraints are simultaneously applied to the inner surface of the wheel hub bore. It is essential to ensure that the midpoint of a single rubber block within the rubber layer lies on the load application plane. This positioning guarantees a more pronounced stress–strain response within the rubber layer when the resilient wheel is subjected to operational loads. Considering the large number of mesh elements and the high computational cost of fully symmetric models, a semi-symmetric resilient wheel model is established by exploiting the wheel’s geometric symmetry. Symmetry constraints are applied along the plane of symmetry to simulate both the press-fitting process and operational conditions, with the symmetric boundary conditions illustrated in Figure 6c. Consequently, when applying straight-line and curved-line operational loads to the semi-symmetrical resilient wheel model, the applied loads should be half the values of the full resilient wheel model. Specifically, this entails applying half the vertical load *F_z_*_1_/2 for straight-line conditions, half the vertical load *F_z_*_2_/2 and half the lateral load *F_y_*_2_/2 for curved-line conditions.

## 3. Analysis of Stress Results and Structural Optimization

### 3.1. Press-Fit Process Stress Analysis

**Figure 7:**
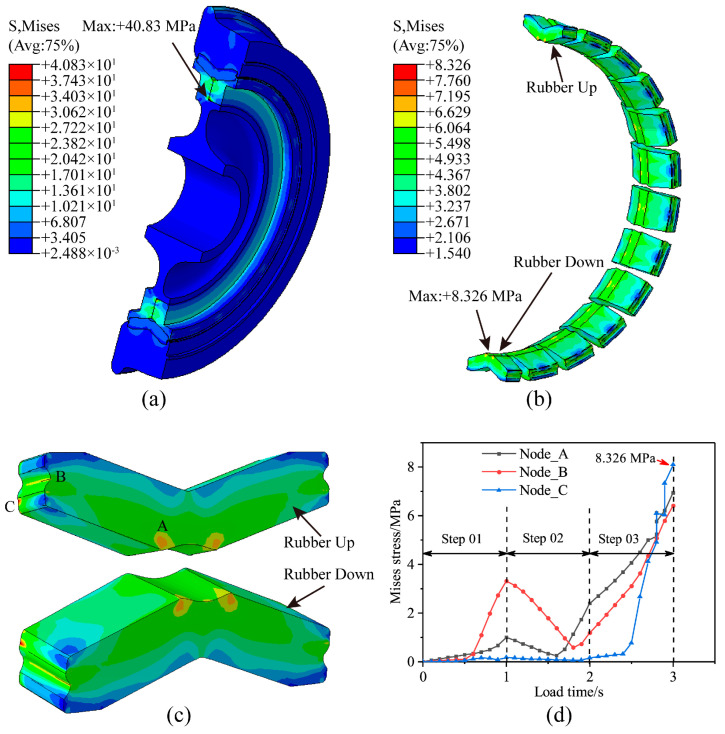
Press-fit stresses in resilient wheels: (**a**) Overall stress contour map of the wheel; (**b**) stress contour map of the rubber layer; (**c**) stress distribution in upper and lower rubber blocks; (**d**) stress evolution in critical regions.

Using the numerical simulation method for the press-fitting process of resilient wheels, the semi-symmetrical wheel model is numerically solved. This produces the stress contour of the resilient wheel after completion of the press-fitting process, as shown in Figure 7a. The maximum global Mises equivalent stress following press-fitting reaches 40.830 MPa, with the peak stress located at the fillet position where the press ring and wheel hub are assembled. The stress contour of the rubber layer in the assembled resilient wheel is presented in Figure 7b. The maximum Mises equivalent stress within the rubber layer reaches 8.326 MPa, with the peak stress occurring at the lateral arc transition of the lower rubber block. The rubber layer of the resilient wheel consists of 26 circumferentially and equidistantly arranged rubber blocks. To illustrate the stress distribution within individual rubber blocks, the stress characteristics of the upper rubber block (Rubber Up) and lower rubber block (Rubber Down) are extracted, as shown in Figure 7c. As indicated, the maximum stresses in the rubber blocks under press-fitting conditions primarily occur at the bottom arc transition point (A) and the side arc transition points (B and C). The variation in Mises equivalent stress in these high-stress regions with respect to the loading history is illustrated in Figure 7d. At a loading time of 1 s (i.e., Step 01), stress peaks occur at positions A and B. This arises because, at this stage, a displacement *U_r_* varying radially is applied to the rubber layer. As depicted in Figure 7b, the rubber undergoes radial displacement, leading to an increase in equivalent stress. As the radial displacement *U_r_* gradually approached zero (i.e., Step 02), the stress values within the rubber block progressively decreased. Concurrently, displacements *U*_zL_/2 and *U*_zR_/2 are applied to the two lateral surfaces of the rubber layer, respectively, causing the stress within the rubber block to exhibit a trend of initially decreasing, followed by an increase. During the loading period of 2–3 s (i.e., Step 03), displacements *U*_zL_ and *U*_zR_ are applied to the two lateral surfaces of the rubber layer, respectively. The Mises equivalent stress within the rubber block increased sharply, with the greatest rate of increase occurring at position C. At *t* = 3 s, the stress reached 8.326 MPa.

### 3.2. Operational Stress Analysis

**Figure 8:**
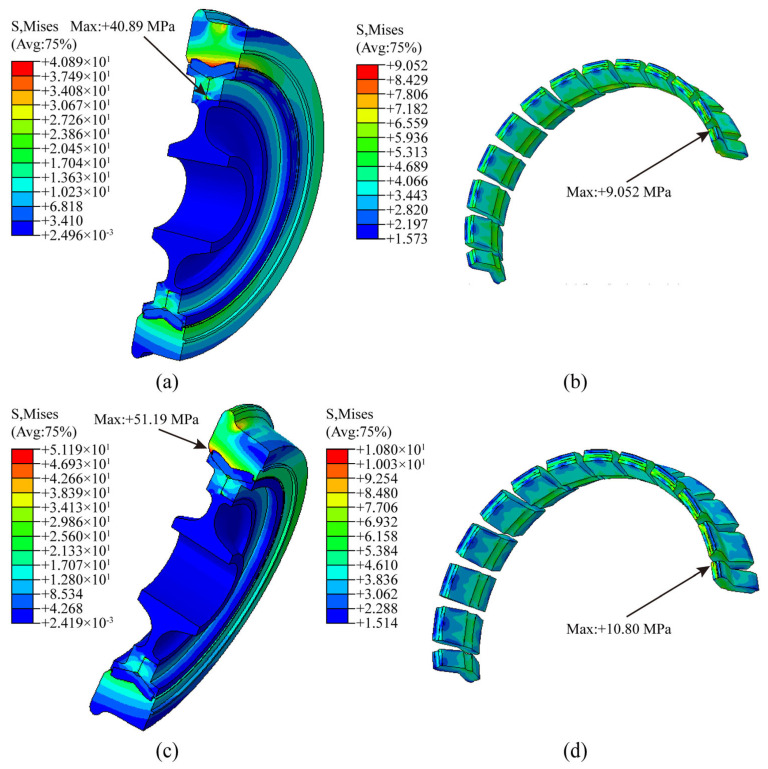
Operational conditions for resilient wheels: (**a**) Stress contour map for wheel under straight-line conditions; (**b**) stress contour map for rubber under straight-line conditions; (**c**) stress contour map for wheel under curved-line conditions; (**d**) stress contour map for rubber under curved-line conditions.

In accordance with the numerical simulation methodology for resilient wheels under operational conditions, linear load (*F_z_*_1_) and curved load (*F*_z2_ and *F*_y2_) are applied to the resilient wheel, corresponding to ABAQUS 2022 finite element analysis steps Step04 and Step05, respectively. The overall stress contour map and rubber layer stress contour map for the resilient wheel under straight-line conditions are shown in Figure 8a,b, respectively. The maximum Mises equivalent stress across the entire wheel reached 40.89 MPa, occurring at the fillet transition between the pressure ring and wheel core. The maximum Mises equivalent stress in the rubber layer is 9.052 MPa, occurring at the lateral fillet transition of the upper rubber block bearing the vertical load (*F_z_*_1_). The stress contour plots for the entire resilient wheel and the rubber layer under curved track conditions are shown in Figure 8c,d, respectively. The maximum Mises stress for the entire wheel reached 51.19 MPa, with the peak equivalent stress occurring at the flange position near the rim side of the wheel rim. The maximum equivalent Mises stress within the rubber layer is 10.80 MPa, with the peak equivalent stress similarly occurring at the side-radius transition of the upper rubber block subjected to vertical and lateral loads (*F*_z2_ and *F*_y2_).

Combining the above simulation results shows that the maximum equivalent stress across the entire resilient wheel assembly under both straight-line and curved-track operating conditions occurs in the non-rubber critical components. Moreover, these maximum equivalent stresses remain below the yield strength of the components (rim yield strength ≥ 600 MPa; hub and pressure ring yield strength ≥ 685 MPa). For both straight-line and curved-track conditions, the maximum equivalent stress in the rubber layer occurs at the lateral arc transition of the upper loaded rubber block. Under curved-track conditions, the maximum stress in the rubber layer reaches 10.76 MPa, with stress peaks concentrated at the fillet transition points of the rubber block. Therefore, by combining the existing dimensional parameters of the critical fillet regions of the rubber block with the equivalent stress results obtained from finite element simulations, optimizing the key structural dimensions of the resilient wheel rubber block to enhance its fatigue resistance and extend its fatigue life represents an effective approach for refining the structural design of the rubber block.

Stress concentration occurs in specific regions characterized by geometric discontinuities, such as fillet transitions, which lead to a local densification of stress flow lines and abrupt changes in structural stiffness. These geometric features promote localized stress amplification. In addition, these regions act as critical nodes in the load transfer path, where loads cannot be uniformly distributed, resulting in localized load accumulation. As a rubber–metal contact interface, frictional effects further intensify stress concentration in these areas. Moreover, the rubber damping component of the resilient wheel is composed of a hyperelastic material. Under the combined influence of geometric and loading factors, the stress concentration regions undergo larger deformations at an early stage. The strain-hardening behavior of rubber leads to an increase in the local elastic modulus, forming a positive feedback mechanism of “deformation–stiffness–stress,” which further exacerbates stress concentration.

Although stress invariants or strain-based parameters are generally considered more appropriate for hyperelastic rubber materials, the present study focuses on a rubber–metal composite component operating under conditions characterized by small-to-moderate deformation and a relatively low degree of multiaxiality. Moreover, the von Mises equivalent stress is a widely accepted criterion for strength assessment and structural optimization of metallic components. The adoption of this metric enables a unified mechanical evaluation framework for both the metal substrate and the rubber component, thereby facilitating coupled optimization across dissimilar materials and enabling consistent comparison of results. Therefore, in this study, the von Mises equivalent stress is employed as the primary indicator for structural optimization and fatigue assessment.

### 3.3. Rubber Components Optimization Methods

Based on the stress analysis results of the rubber block during the resilient wheel press-fitting process and under operational conditions, the maximum equivalent stress is concentrated at the rounded transition points on the bottom and sides of the rubber block. Therefore, structural optimization research can be conducted on the dimensions of these rounded transition points. This study employs the Isight 2022 module within ABAQUS 2022 finite element software. By automatically iteratively modifying the rubber block’s dimensions, remeshing the model, and solving for the maximum stress value *S*_max_ within the rubber layer, the optimal rubber block dimensions corresponding to the minimum (*S*_max_)_min_ under identical operational conditions are determined. This achieves an automated optimization process for the critical dimensional regions of the rubber block.

**Figure 9:**
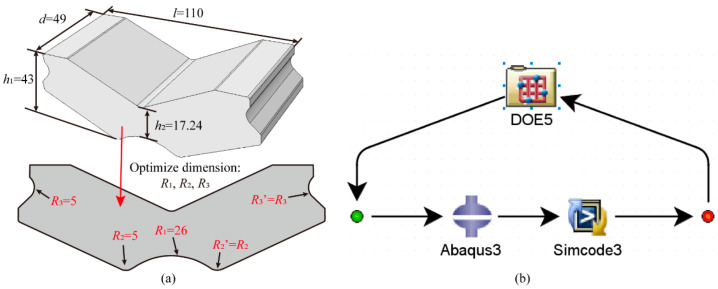
Rubber structure dimensional optimization: (**a**) Critical dimensions of rubber blocks; (**b**) Isight 2022 optimization solution workflow.

The overall structural dimensions of the resilient wheel rubber block are shown in Figure 9a. The total length *l* is 110 mm, height *h*_1_ is 43 mm, depth *d* is 49 mm, and the height *h*_2_ of the central rubber block region is 17.24 mm. The regions experiencing higher equivalent stresses during the press-fitting process and operational condition simulation are related to parameters *R*_1_, *R*_2_, and *R*_3_. Therefore, *R*_1_, *R*_2_, and *R*_3_ can be selected as the optimization parameters for the rubber block. The initial values for the bottom corner radii are *R*_1_ = 26 mm and *R*_2_ = 5 mm, while the side corner radius is *R*_3_ = 5 mm. Given the block’s symmetrical structure, the conditions *R*_2_ = *R*_2_′ and *R*_3_ = *R*_3_′ are satisfied.

When employing the Isight 2022 module for structural optimization studies of rubber blocks, it is necessary to insert the Abaqus 2022 solver component into the workflow diagram to solve the finite element model of the rubber block structure after each dimensional update. Subsequently, the Simcode component calculates the maximum Mises equivalent stress values within the rubber layer from the results. Thereafter, the structural dimensions of the rubber block are updated via the DOE module component, with the optimization workflow illustrated in Figure 9b. Within the DOE (Design of Experiments) module, the optimization technique selected is Full Factorial. This specifies the number of levels for each factor, investigating all combinations of all factors across all levels. The advantage of this approach lies in its ability to evaluate all *p*-1 order effects for *p* levels, enabling assessment of all possible factor interactions. The initial dimension ranges for this rubber structure optimization are set as follows: *R*_1_ [24 mm, 25 mm, 26 mm, 27 mm, 28 mm]; *R*_2_ [3 mm, 4 mm, 5 mm, 6 mm, 7 mm]; *R*_3_ [3 mm, 4 mm, 5 mm, 6 mm, 7 mm]. The combined ranges of *R*_1_, *R*_2_, and *R*_3_ form the design matrix for this structural optimization. By iteratively calculating the maximum Mises equivalent stress *S_i_*_max_ across 125 distinct combinations of these parameters, the optimal solution is identified as the set of *R*_1_, *R*_2_, and *R*_3_ values yielding the minimum *S_i_*_max_.

**Figure 10:**
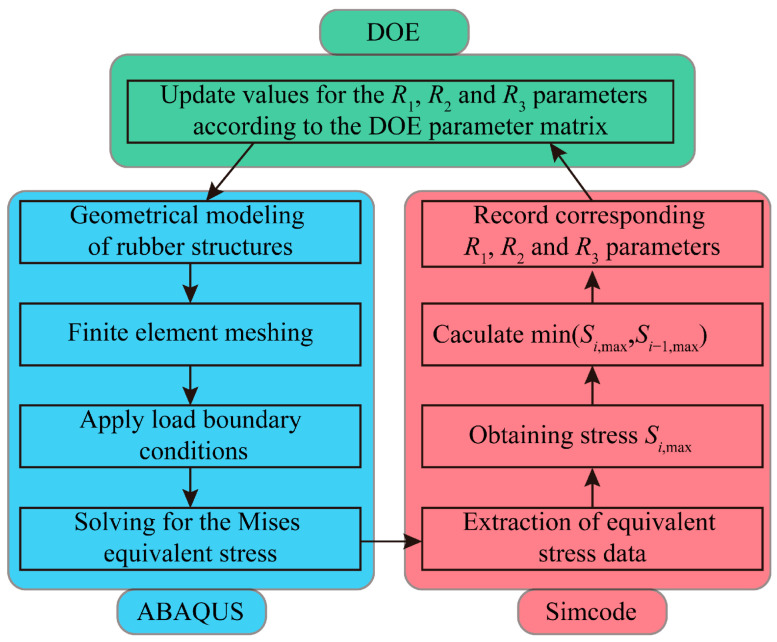
Rubber structure optimization cycle flowchart.

The detailed rubber structure optimization process is illustrated in Figure 10. First, the initial rubber structure geometry with dimensions *R*_1_ = 26 mm, *R*_2_ = 5 mm, and *R*_3_ = 5 mm is imported into the ABAQUS 2022 component. A finite element mesh is then generated, and load boundary conditions are applied. Finally, ABAQUS 2022 is invoked to solve for the Mises equivalent stress of the resilient wheel rubber model. Subsequently, within the Simcode module, a custom Python 2.7.0 script is invoked to extract stress data from the .odb structure. The maximum equivalent stress *S_i_*_max_ for the current model is obtained and compared with the maximum equivalent stress *S_i_*_−1max_ from the previous calculation to derive the smaller value (*S_i_*_max_, *S_i_*_−1max_)_min_ (if this is the first iteration, no comparison is required). Finally, within the DOE component, the parameter value matrix for *R*_1_, *R*_2_, and *R*_3_ is invoked to update the critical parameter values of the rubber block. These parameters are then passed to the ABAQUS 2022 component to update the rubber block geometry, initiating the subsequent Mises equivalent stress calculation for the rubber block. Through iterative computation over 125 cycles, the minimum value (*S_i_*_max_)_min_ for the rubber block is obtained, along with the optimal parameter values for *R*_1_, *R*_2_, and *R*_3_ corresponding to this minimum value.

### 3.4. Structural Optimization Results

Based on the key parameters *R*_1_, *R*_2_, and *R*_3_ determined using the Isight 2022 software for the rubber vibration-damping element, a model of the rubber vibration-damping element with specified *R*_1_, *R*_2_, and *R*_3_ parameters is established in ABAQUS 2022. Load boundary conditions corresponding to the curved operating condition from Section 3.2 are applied, and the maximum equivalent stress value *S_i_*_max_ for the rubber vibration-damping element is calculated. Different values for these key parameters correspond to distinct operating conditions. All parameter values and the calculated equivalent stress values are presented in Table 2, Table 3, Table 4, Table 5 and Table 6, respectively. Through iterative calculations using Isight 2022 software, the critical structural dimensions of the rubber element corresponding to the minimum equivalent stress are determined. As shown in Table 2, Table 3, Table 4, Table 5 and Table 6, the minimum equivalent stress of the rubber vibration-damping element is 8.86 MPa, with the corresponding critical structural dimensions *R*_1_, *R*_2_, and *R*_3_ being 28 mm, 4 mm, and 7 mm, respectively.

## 4. Fatigue Life Prediction Research and Comparison

The resilient wheel rubber block is fabricated from hyperelastic material, capable of withstanding large strains while exhibiting outstanding properties such as vibration damping, noise reduction, and sealing. During fatigue crack propagation, the crack growth rate satisfies a power-law relationship with tear energy. As tear energy increases, the crack gradually propagates; when the crack size reaches a critical length, ultimate failure occurs. Tear energy theory constitutes a fatigue analysis methodology for rubber, integrating fracture mechanics principles with the *S*-*N* curve approach. It serves to predict the fatigue life and crack initiation direction of rubber materials. This study investigates the fatigue life prediction of resilient wheel rubber vibration-damping components using the Fe-safe 2022 module within the finite element software ABAQUS 2022. It further compares the life differences before and after structural optimization of the rubber block.

### 4.1. Rubber Structural Life Prediction Method

The fatigue life of rubber structures primarily comprises crack initiation life and crack propagation life. Fatigue crack nucleation occurs at stress or strain concentration points within the rubber material, or at the interface between particles and the matrix. The crack propagation life generally equates to the total fatigue life of the rubber structural component. Assuming a rubber block structure harbors a microcrack of initial length *c*_0_ prior to cyclic loading, the repeated application of cyclic fatigue loads causes the fatigue crack to propagate continuously. This process releases strain energy whilst generating tear energy. When the fatigue crack reaches a critical length *c_f_*, the rubber structure is deemed to have failed due to fatigue. Consequently, the number of load cycles required for the crack to propagate from its initial length *c*_0_ to the critical length *c_f_* can be defined as the fatigue life of the rubber material. This is expressed as:

(3) $$N_{f}=\int_{c_{0}}^{c_{f}}\frac{1}{f(T(c,t))}dc$$

where *N_f_* denotes fatigue life; *T*(*c*, *t*) represents the fracture energy, expressed as a function of the current crack length *c* and time *t*; and *f*(*T*(*c*, *t*)) constitutes the expression for the crack propagation model. During the fatigue crack nucleation stage, fracture energy may be represented in the form of fracture toughness, namely

(4) $$T=2\pi W_{c}c$$

where *T* denotes tear energy, and *W_c_* denotes fracture energy. During the fatigue crack propagation stage, the fatigue crack propagation rate of rubber is governed by equivalent tear energy. Therefore, substituting the equivalent tear energy for the stress intensity factor range in the Paris formula yields the crack propagation rate equation. The equivalent tear energy is expressed as:

(5) $$T_{eq}={T_{\max}}^{\frac{F(R)}{F(0)}}{T_{c}}^{\frac{F(0)-F(R)}{F(0)}}$$

(6) $$F(R)=F_{0}e^{F\exp R}+F_{1}R+F_{2}R^{2}+F_{3}R^{3}$$

Here, *T_eq_*, *T*_max_ and *T_c_* denote the equivalent, maximum and critical tear energies, respectively; *F* is a function of the stress ratio *R*; and *F*_exp_, *F*_0_, *F*_1_, *F*_2_ and *F*_3_ represent material parameters. When calculating the fatigue life of rubber structural components using Fe-safe 2022 software, the corresponding material fatigue parameters must be entered into the material parameter table. The fatigue parameters for rubber materials are shown in Table 7.

### 4.2. Calculation of Service Life for Resilient Wheels

**Figure 11:**
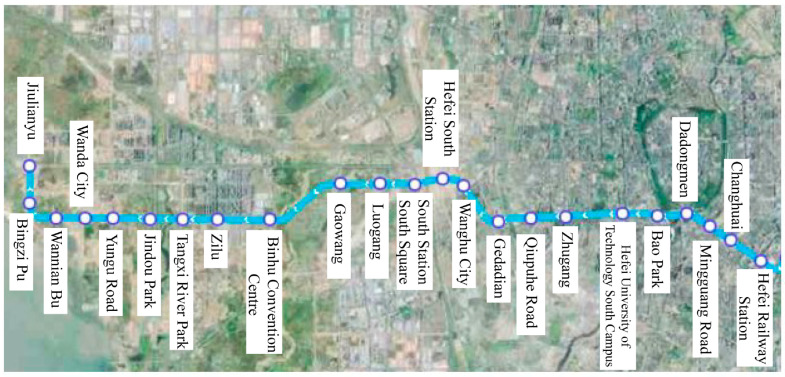
Operational route of Hefei Metro Line 1.

According to the fatigue life prediction method for rubber structures, the load cycle count corresponding to the resilient wheel reaching its fatigue damage threshold can be calculated. This corresponds to the rotational revolutions of the resilient wheel during its service life (one wheel revolution is considered one load cycle). When assessing the fatigue life of rolling stock structures, the number of load cycles endured by the structure should be converted into the corresponding actual operational mileage or operational days on the track. Taking Hefei Metro Line 1 in China as an example, this paper estimates the operational mileage under straight-line and curved-line conditions, as illustrated in Figure 11. The line’s total length *S* is 24.58 km, featuring 23 stations with operating hours from 6:30 to 22:40,which can been shown as Table 8. The line deploys *n* = 21 operational vehicles daily, with *N* = 350 train trips (each trip equating to one operational kilometer) throughout the day. Vehicle routes are categorized solely as straight or curved, with only sections featuring curve radii less than 800 m classified as curved sections for statistical purposes. Given that certain sections between Jiulianwei and Hefei Railway Station feature larger curve radii (exceeding 1200 m) and shorter lengths, exerting minimal influence on acceleration and stress signals, the table exclusively lists conditions with curve radii below 1200 m. The straight track mileage *S_straight_* can be derived by subtracting the curved track mileage *S_curve_* from the total mileage.

Based on the curve representation data from Jiulianwei to Hefei Railway Station, sections with a curve radius less than 800 m are classified as curved zones. Calculations indicate that the curved mileage *S_curve_* for a single mileage segment is 4.71 km. Therefore, the straight-line mileage *S_straight_* can then be calculated using Equation (7).

(7) $$S_{straight}=S-S_{curve}=20.29\mathrm{km}$$

Therefore, the average straight-line mileage and curved mileage traveled by each operational metro train within a day are calculated using Equation (8) and Equation (9), respectively.

(8) $$S_{d,straight}=2*\frac{N}{n}*S_{straight}=676.33\mathrm{km}$$

(9) $$S_{d,curve}=2*\frac{N}{n}*S_{curve}=157.00\mathrm{km}$$

Therefore, based on the nominal rolling radius of the resilient wheels being *R_W_* = 0.37 m, it can be calculated that during a day’s operation of the metro vehicle, the number of revolutions *m_straight_* completed by the resilient wheels under straight-line conditions and the number of revolutions *m_curve_* completed under curved conditions are determined, respectively, by Equations (10) and (11).

(10) $$m_{straight}=\frac{S_{d,straight}}{2\pi R_{W}}=290924$$

(11) $$m_{curve}=\frac{S_{d,curve}}{2\pi R_{W}}=67534$$

The stress–strain results for the rubber vibration-damping elements, calculated separately for straight-line and curved-line operating conditions for the resilient wheels, can be imported into ABAQUS’s fatigue life calculation module, Fe-safe 2022, to compute fatigue life. Given the number of revolutions required for straight-line and curved-line conditions across one day’s operation of the resilient wheel, these revolutions can be separately configured within Fe-safe 2022 software. This enables the software to directly calculate the operational days of the resilient wheel, thereby determining its actual service life under real-world track conditions.

### 4.3. Fatigue Life Comparison Before and After Structural Optimization

To conduct fatigue life analysis on resilient wheel rubber vibration damping components using the Fe-safe 2022 module within ABAQUS 2022, it is first necessary to obtain stress–strain data for these components under specified loading conditions. Subsequently, the .odb file generated by the ABAQUS 2022 solver is imported into the Fe-safe 2022 software module. By selecting an appropriate fatigue life calculation method and inputting key model parameters, the service life of the rubber damping components can be predicted. The Fe-safe 2022 software incorporates the Endurica fatigue algorithm, which comprehensively accounts for the influence of multiple physical, chemical, and mechanical properties on rubber fatigue life. Consequently, this method is widely employed for fatigue life assessment across various types of rubber materials.

**Figure 12:**
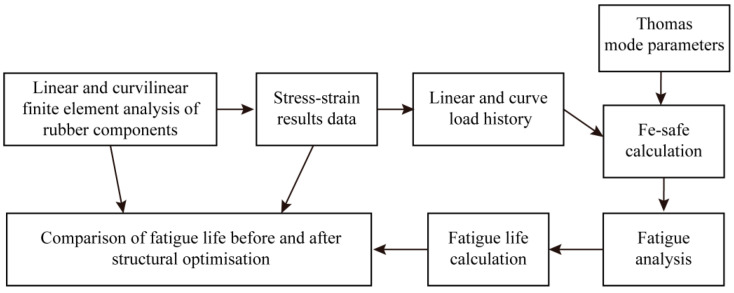
Calculation process for service life before and after optimization of rubber components.

The rubber fatigue crack propagation model employed herein establishes the relationship between tear energy and crack growth velocity. Subsequently, based on the operational conditions of resilient wheels on actual track networks, the fatigue life of elastic rubber vibration-damping components is determined using track-specific data, calculated according to the number of straight-line and curved-track operating cycles per day as specified in Section 4.2. Subsequently, fatigue life calculations are performed for the rubber components under actual operational track conditions (encompassing both straight-line and curved load scenarios), both before and after structural optimization. The differences in predicted life between the pre- and post-optimization stages are then compared. The entire life calculation process and methodology are illustrated in Figure 12.

**Figure 13:**
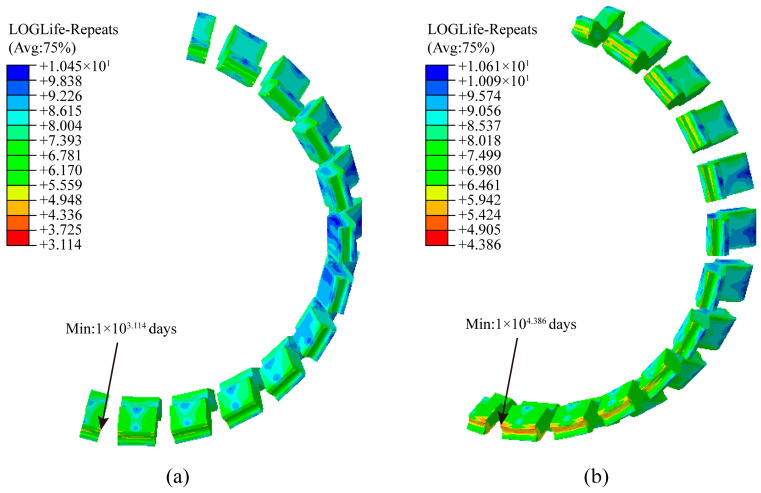
Comparison of fatigue life counters for rubber components: (**a**) Before optimization; (**b**) after optimization.

The .odb result files for linear and cyclic loading conditions, solved using the finite element software ABAQUS 2022, are imported into the Fe-safe 2022 software. Corresponding stress–strain data is extracted for fatigue life calculations. The rubber material model is selected and assigned the crack propagation parameters from Table 7. Simultaneously, stress–strain data extracted post-press-fit, along with stress–strain data for both linear and curved load conditions, are combined to form load history data for linear and curved loading cycles. Within a single load block, the number of cycles per day for both straight-line *m_straight_* and curved-line *m_curve_* loading cycles is specified. Thus, each load block cycle computed in Fe-safe 2022 represents the combined total of straight-line and curved-line cycles for one day of metro vehicle operation. Finally, the Endurica algorithm, suitable for rubber fatigue life calculations, is selected to solve for the fatigue operational life of the resilient wheel’s rubber vibration-damping element, yielding the operational days of the rubber vibration-damping element under actual operational line conditions. Comparing the fatigue resistance of resilient wheels based on pre- and post-optimization fatigue life calculations for the rubber vibration-damping elements, as shown in Figure 13, reveals that the minimum fatigue life of the rubber element prior to structural optimization is 1 × 10^3^ days (i.e., 1300 days). Post-optimization, the minimum fatigue life increased to 1 × 10^3^ days (i.e., 24,322 days). The minimum fatigue life positions for both pre- and post-optimization rubber components are predominantly located at the lateral arc transition points of the rubber element. Consequently, structural optimization of the rubber vibration-damping component significantly enhanced its fatigue life and fatigue resistance.

## 5. Conclusions

This study presents a comprehensive mechanical simulation and fatigue life assessment of rubber vibration-damping components in resilient wheels for rail vehicles. A three-dimensional finite element (FE) model of the resilient wheel is developed to characterize the stress–strain response during the press-fitting assembly process. Load cases corresponding to straight-line and curved-track operating conditions are defined in accordance with EN 13979-1 and UIC 510-5 standards, and the resulting stress distributions in both the rubber damping element and associated wheel components are systematically evaluated.

Based on the identified high-stress regions within the rubber component, structural optimization is carried out using a Design of Experiments (DOE) approach implemented in the Isight 2022 optimization platform. This process yields an optimized set of geometric parameters for the rubber vibration-damping element. Furthermore, a fatigue life prediction methodology is established by integrating a rubber crack propagation model with actual service mileage data of resilient wheels. Fatigue life assessments are performed using Fe-safe 2022 software, enabling a comparative analysis of the rubber damping components before and after structural optimization. The main conclusions of this study are summarized as follows:

The maximum global Mises equivalent stress of the resilient wheel after press-fitting is 40.830 MPa, with the peak stress located at the fillet transition between the press ring and wheel core during press-fitting. The maximum Mises equivalent stress within the rubber layer of the resilient wheel post-press-fitting is 8.326 MPa, with the stress peak occurring at the lateral arc transition of the lower rubber block in the rubber layer.

Under straight-line operating conditions, the maximum global Mises equivalent stress across the entire resilient wheel reaches 40.89 MPa, occurring at the fillet transition between the press ring and wheel core. The maximum Mises equivalent stress within the rubber layer is 9.052 MPa, appearing at the lateral fillet transition of the upper rubber block that bears the vertical load (*F*_z1_).

Under curved-track operating conditions, the maximum global Mises stress of the resilient wheel reaches 51.19 MPa, with the peak equivalent stress located at the flange near the rim side of the wheel. The maximum Mises equivalent stress within the rubber layer is 10.80 MPa, occurring at the fillet transition on the side of the upper rubber block subjected to both vertical (*F*_z2_) and lateral (*F*_y2_) loads.

The initial critical high-stress region dimensions for the resilient wheel’s rubber vibration-damping element are *R*_1_ = 26 mm and *R*_2_ = 5 mm, with the rubber block’s side fillet radius *R*_3_ set at 5 mm. Following structural optimization, the critical dimensions *R*_1_, *R*_2_, and *R*_3_ are adjusted to 28 mm, 4 mm, and 7 mm, respectively.

The minimum fatigue life of the rubber vibration-damping element prior to structural optimization is 1300 days, whereas the minimum fatigue life after optimization reaches 24,322 days. Structural optimization markedly extends the fatigue life of the rubber vibration-damping element and enhances its fatigue resistance performance.

## Figures and Tables

**Table 2:** Optimization process and stress results for parameter *R*_1_ set to 24.

Cases	1	2	3	4	5	6	7	8	9	10	…	25
*R*_1_/mm	24	24	24	24	24	24	24	24	24	24	…	24
*R*_2_/mm	3	3	3	3	3	4	4	4	4	4	…	7
*R*_3_/mm	3	4	5	6	7	3	4	5	6	7	…	7
*S_i_*_max_/MPa	10.64	10.24	9.43	9.82	9.28	10.02	10.01	9.81	9.55	9.44		9.14

**Table 3:** Optimization process and stress results for parameter *R*_1_ set to 25.

Cases	26	27	28	29	30	31	32	33	…	35	…	50
*R*_1_/mm	25	25	25	25	25	25	25	25	…	25	…	25
*R*_2_/mm	3	3	3	3	3	4	4	4	…	5	…	7
*R*_3_/mm	3	4	5	6	7	3	4	5	…	7	…	7
*S_i_*_max_/MPa	10.42	10.20	9.52	9.26	9.64	10.39	9.96	9.60	…	9.05		9.19

**Table 4:** Optimization process and stress results for parameter *R*_1_ set to 26.

Cases	51	52	53	54	55	56	57	58	59	60	…	75
*R*_1_/mm	26	26	26	26	26	26	26	26	26	26	…	26
*R*_2_/mm	3	3	3	3	3	4	4	4	4	4	…	7
*R*_3_/mm	3	4	5	6	7	3	4	5	6	7	…	7
*S_i_*_max_/MPa	10.73	9.96	9.48	9.40	9.56	10.30	10.39	9.52	9.33	8.92		9.02

**Table 5:** Optimization process and stress results for parameter *R*_1_ set to 27.

Cases	76	77	78	79	80	81	82	83	84	85	…	100
*R*_1_/mm	27	27	27	27	27	27	27	27	27	27	…	27
*R*_2_/mm	3	3	3	3	3	4	4	4	4	4	…	7
*R*_3_/mm	3	4	5	6	7	3	4	5	6	7	…	7
*S_i_*_max_/MPa	11.36	10.32	9.44	9.68	9.31	10.89	10.35	9.76	9.02	9.20		9.04

**Table 6:** Optimization stress results for parameter *R*_1_ set to 27 with optimal solution (case 110).

Cases	101	102	103	104	105	106	107	108	109	110	…	125
*R*_1_/mm	28	28	28	28	28	28	28	28	28	28	…	28
*R*_2_/mm	3	3	3	3	3	4	4	4	4	4	…	7
*R*_3_/mm	3	4	5	6	7	3	4	5	6	7	…	7
*S_i_*_max_/MPa	10.98	10.30	9.46	9.65	9.33	9.45	9.33	9.01	9.08	8.86		9.09

**Table 7:** Fatigue parameters of rubber materials.

Initial Length *c*_0_/mm	Critical Length *c_f_*/mm	Critical Tear Energy *T_c_*/(J/mm^2^)	Critical Crack Propagation Rate *r_c_*/(mm/Cycle)	*F*_0_	*F*_1_	*F*_2_	*F*_3_
0.00933	1.000	33,890	4.73625 × 10^−3^	2.717	25.310	−102.70	164.250

**Table 8:** Curve information from Jiulianwei to Hefei railway station.

Interval	Boundary Mileage/m	Curve Radius *R*/m	Curve Length *L*/m
Bingzi Pu–Wannian Bu	K27 + 625.354	380	646.903
Wannian Bu–Wanda City	K26 + 452.644	1000	127.748
	K26 + 300.843	1000	127.748
Wanda City–Yungu Road	K25 + 183.908	200	22.131
Yungu Road–Jindou Park	K24 + 932.943	1200	85.036
	K24 + 856.397	1200	85.036
	K24 + 433.674	450	175.597
	K24 + 225.632	455	177.150
Jindou Park–Tangxi River Park	K23 + 580.089	995	121.911
	K23 + 369.488	450	162.304
	K23 + 173.835	645	142.202
Tangxi River Park–Zilu	K22 + 904.289	1200	92.165
	K22 + 788.530	1205	92.474
	K22 + 76.134	1205	92.474
Zilu–Binhu Convention Centre	K21 + 700.893	1205	92.474
	K20 + 667.112	1195	91.857
Binhu Convention Centre–Gaowang	K20 + 296.495	350	320.967
	K20 + 101.322	800	114.608
	K19 + 372.961	1000	119.695
	K18 + 888.015	450	439.142
Gaowang—Luogang	K17 + 454.236	1200	101.91
	K17 + 267.770	800	155.152
South Station South Square—Hefei South Railway Station	K15 + 753.160	350	213.837
	K15 + 496.309	350	169.051
Hefei South Railway Station—Wanghu City	K14 + 718.249	300	352.372
Wanghu City—Gedadian	K14 + 122.202	350	113.364
	K13 + 544.599	450	316.968
	K13 + 178.269	350	454.047
Gedadian—Qiupuhe Road	K12 + 402.548	1200	342.579
Zhugang—Hefei University of Technology South Campus	K10 + 524.56	800	148.705
	K10 + 206.074	1000	135.951
Hefei University of Technology South Campus—Bao Park	K8 + 743.151	800	146.425
	K8 + 140.096	350	136.077
Bao Park—Dadongmen	K7 + 899.658	300	301.202
	K7 + 598.456	350	337.847
	K7 + 243.753	800	116.438
Dadongmen—Mingguang Road	K6 + 980.918	350	227.511
Changhuai—Hefei Railway Station	K4 + 677.324	Crossroad 406	
	K4 + 542.324	Crossroad 402	

K denotes kilometers in whole numbers; for example, K27 + 625.354 indicates a distance of 27,625.354 m.

## Data Availability

The original contributions presented in this study are included in the article. Further inquiries can be directed to the corresponding author.
